# Association Between the Type of Dental Care Setting and the Risk of Dental Implant Failure in Korea: A Retrospective Nationwide Population-Based Cohort Study

**DOI:** 10.34172/ijhpm.9238

**Published:** 2025-12-28

**Authors:** Yu-Rin Kim, Seon-Rye Kim, Minkook Son

**Affiliations:** ^1^Department of Dental Hygiene, Silla University, Busan, Republic of Korea.; ^2^Institute of Health Medical Education Convergence Research, Kangwon National University, Gangwon-do, Republic of Korea.; ^3^Department of Physiology, Dong-A University College of Medicine, Busan, Republic of Korea.; ^4^Department of Data Sciences Convergence, Dong-A University Interdisciplinary Program, Busan, Republic of Korea.

**Keywords:** Dental Clinics, Dental Implantation, Dental Restoration Failure, Insurance, Korea

## Abstract

**Background:**

In Korea, the introduction of reimbursable dental implant procedures has intensified competition among dental practices, albeit with an increase in consumer complaints related to implant failures. Our study aimed to evaluate the association between the dental implant failure risk and the dental care setting.

**Methods:**

This retrospective cohort study used data from the Health Screening Cohort (HEALS) of the Korean National Health Insurance Service (NHIS) to analyze the risk of dental implant failure from January 1, 2016, to December 31, 2019. The risk of dental implant failure according to the dental care setting was assessed using inverse probability of treatment weighting (IPTW) adjusted Cox regression analysis. The covariates included demographic, socioeconomic, and clinical factors. The hazard ratios (HRs) with 95% confidence intervals (CIs) were estimated. Among 44 220 cases, additional analyses were performed by stratifying implant procedures in private dental practice (n = 40 502) into quartiles to assess the risk of failure according to procedural volume.

**Results:**

Hospital-based dental clinics exhibited a lower implant failure risk compared with private dental practices (HR: 0.35, 95% CIs: 0.30–0.41) and group dental practices (HR: 0.34, 95% CIs: 0.20–0.58). Private dental practices with the top 10% and 5% procedural volume showed a higher failure risk (HR: 1.23, 95% CIs: 1.09–1.38; HR: 1.38, 95% CIs: 1.23–1.54, respectively) relative to practices handling the remaining 90% and 95%.

**Conclusion:**

The risk of dental implant failure was lower in hospital-based dental clinics compared with private and group dental practices, indicating the need for more systematic and thorough postoperative care to improve implant safety in settings associated with higher failure risk.

## Background

**Key Messages**

**Implications for policy makers**

- Competition among dental practices in Korea may have a deleterious effect on patient care, particularly for older adults. As the correlation between dental care setting and implant failure has been identified, systematic procedures and rigorous postoperative management are essential to reduce the risk of implant failure.
- To address this issue, implementing regulations on the number of daily procedures performed by dentists, especially for elderly patients, individuals with complications, or those requiring high-difficulty surgeries, should be considered. Such measures would enable more detailed treatment planning and careful surgical execution.
- Ultimately, ensuring adequate case analysis and individualized treatment within appropriate dental care settings may enhance patient safety, reduce implant failure rates, and improve the overall quality of dental healthcare delivery.

**Implications for the public**

This study identified a significant association between the dental care setting and the risk of dental implant failure, with lower failure rates observed in hospital-based dental clinics compared with private dental practices and group dental practices. Therefore, intense price competition to attract patients requiring implants, which is common in private and group dental practices, often leads individuals to low-cost settings that provide minimal per-patient resources, a pattern that may increase implant failure rates and ultimately shift the risk and burden to patients. Patients should consider not only treatment volume but also the complexity of the procedure, the clinician’s experience, and the resources available within the setting to potentially help mitigate the risk of implant failure.

In Korea, dental implants are covered by health insurance; thus, patients only have to pay 30% of the total cost, and the number of patients receiving this procedure is increasing every year.^1^ Osseointegrated dental implants are a reliable treatment option for patients with complete or partial edentulism, with success rates of 97% over 10 years and 75% over 20 years.^2,3^ Nonetheless, individualizing the treatment protocol is important for a good prognosis and patient satisfaction, and potential risk factors for dental implant failure are of increasing interest.

 The prevalences of early and late failure are 0.5%–5.2% and 0.5%–7.8%, respectively,^4,5^ and the weighted average survival rate after re-implantation following failure is 86.3%, which is very low compared to the initial implant survival rate.^6^ Dental re-implantation also complicates the treatment process, prolongs the treatment period, and jeopardizes the efforts of dentists to achieve satisfactory function and esthetics. In addition, it usually involves additional costs and procedures for the patients. Accordingly, the number of applications for damage relief related to dental implant procedures received by the Korea Consumer Agency is increasing every year. The principal reasons for the applications included side effects related to dental implant surgery and contract-related complaints, such as a refund of prepaid medical expenses.^7^ Despite the continued occurrence of implant-related damage, in-depth investigations exploring the association between dental implant failure and dental care settings are lacking.

 Since the pandemic, the number of patients visiting dental care settings in Korea decreased by 35%, diminishing the latter’s income by 34%. Consequently, approximately 10% of dentists are considering closing their practices,^8^ Notably, up to 80% of dentists subjectively report feeling competition.^9^ Therefore, the competition for reimbursed dental implant procedures is intensifying in the dental industry, as is the competition to attract patients to dental care settings. The Korea Consumer Agency reported that side effects and refund-related damages are relatively more common in low-cost dental implant procedures and urged people to be wary of dental medical institutions that offer excessive event discounts and require prepayment of the entire procedure fee when contracting for dental implant procedures.^10^ Thus, it is important to choose a dental care setting that can provide follow-up care even after dental implant placement. Understanding whether the risk of dental implant failure varies depending on the dental care setting could help manage and improve the quality of procedures and patient safety.

 To date, most studies related to dental implant failure have analyzed the causes of failure^11-13^ and investigated the risk factors for dental implant complications.^14-16^ Studies analyzing the risk of dental implant failure across different dental care settings are lacking. Moreover, no studies have conducted volume–outcome analyses in private dental practices, where the majority of dental implant procedures are currently performed. Therefore, this study investigated the association between the risk of dental implant failure in private dental practices, group dental practices, and hospital-based dental clinics using the Health Screening Cohort (HEALS) within the National Health Insurance Service (NHIS).

## Materials and Methods

###  Data Sources

 We conducted a population-based, retrospective, cohort study using NHIS-HEALS data from January 1, 2016, to December 31, 2019. Data were extracted and de-identified for research purposes, including the patients’ demographic characteristics, diagnoses, prescribed medications, non-surgical and surgical treatments, and medical facilities such as claims.^17^ Further details regarding the NHIS-HEALS database are available at: https://nhiss.nhis.or.kr/en/z/a/001/lpza001m01en.do. The NHIS-HEALS database is a publicly available anonymous dataset, but analysis requires government approval; thus, the need for informed consent was waived. This study was conformed to the Strengthening the Reporting of Observational Studies in Epidemiology (STROBE) guidelines.^18^

###  Study Population

**Figure 1:**
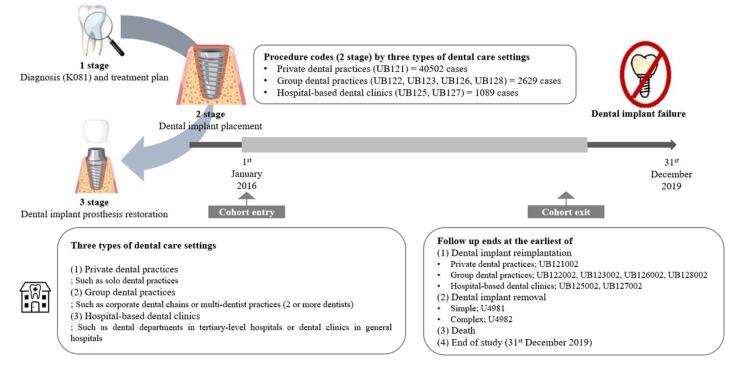
Flow of Research Methods.

 This study included only cases of dental implantation performed from January 1, 2016, and information prior to this date was only used to verify the eligibility criteria and medical history. The International Classification of Diseases, 10th revision (ICD-10) code for reimbursable dental implants is K081, and the specific procedure codes are presented in Table S1 (Supplementary file 1). In this study, dental care settings were categorized into private dental practice, group dental practice, and hospital-based dental clinics. We defined a private dental practice as an independently operated dental office managed by a single dentist. A group dental practice was defined as a clinic where two or more dentists jointly provided care within a shared facility. Hospital-based dental clinics were defined as dental services affiliated with tertiary care hospitals, general hospitals, or university hospitals, where multidisciplinary and advanced treatments are typically available. For more accurate case extraction, oriental medicine hospitals and public health centers that did not match the dental care setting and specific procedures were excluded. The final analysis included 44 220 cases that had completed the second stage of the implant procedure. The cases were followed up from the day of dental implant placement to the day of incidence of dental implant failure, day of death, or December 31, 2019, whichever occurred first (Figure 1).^19,20^

###  Study Outcomes

 Table S1 defines the dental implant failure types for this study. Definition 1 (Re-implantation): Among cases of reimbursable dental implant fixture placement, procedure codes were reviewed according to the dental care setting for cases in which re-implantation was performed due to osseointegration failure. Definition 2 (Removal surgery): Cases in which dental implant removal surgery was performed were designated to one of two categories: simple removal or complex removal. Simple removal was defined as the removal of a dental implant fixture when osseointegration had failed. In contrast, complex removal was defined as the removal of a fixture without mobility, which required the use of a trephine burr or dedicated removal kit, due to concerns such as fixture fracture or potential nerve injury. Dental implant re-implantation (Definition 1) can only be performed for reimbursable dental implant recipients, whereas dental implant removal surgery (Definition 2) can be performed for reimbursable and non-reimbursable dental implant recipients.^19,20^

###  Covariates

 Clinical variables were extracted from the NHIS database to explore the baseline characteristics of the study population based on the date of each participant’s first dental implant placement (stage 2). The extracted variables included demographics (sex, age, income level, disability level, and residential area) and major non-communicable diseases, such as hypertension (I10, I11), diabetes (E10–E14), dyslipidemia (E78), and osteoporosis (M81). The Charlson Comorbidity Index (CCI)^21^ was calculated by considering pre-existing conditions within 1 year using the ICD-10 code. Health screening variables included body mass index, systolic blood pressure, diastolic blood pressure, fasting glucose, hemoglobin, glomerular filtration rate, smoking, alcohol consumption, and regular exercise.

###  Statistical Analyses

 Differences in covariates among the three groups were compared using the analysis of variance and chi-square test. The incidence of dental implant failure in the three groups was measured in units of 1000 person-years during the follow-up period. A Kaplan–Meier curve was generated to analyze the risk of dental implant failure, followed by the log-rank test. To enhance comparability of risk across dental institutions, we employed the inverse probability of treatment weighting (IPTW) method.^22^ Stabilized inverse probability weights were derived from propensity scores estimated using logistic regression to estimate the population average treatment effects while maintaining optimal covariate balance among the groups. The covariates considered included age, sex, income level, disability, residence, hypertension, diabetes, dyslipidemia, osteoporosis, CCI, smoking, alcohol consumption, regular exercise, body mass index, hemoglobin level, and glomerular filtration rate. Covariate balance was evaluated using standardized differences, with values >0.1 indicating imbalance; all covariates exhibited standardized difference values below this threshold. Furthermore, Figure S1 depicts the distributions of the propensity scores before and after IPTW, confirming adequate overlap across groups. Subsequently, the Cox proportional hazards model was used to compute the hazard ratios (HRs) and 95% confidence intervals (CIs) to analyze the risk of dental implant failure. The proportional hazard assumption was evaluated using the Schoenfeld residuals test with the logarithm of cumulative hazards function based on the Kaplan–Meier estimates. There was no interference with the assumption of the proportional hazard risk over time. Additionally, to assess the volume–outcome relationships, the risk of failure was analyzed by procedural volume quartiles, restricted to private dental practices, accounting for more than 90% of all procedures. The NHIS dataset was cleaned prior to analysis. Duplicate records, missing values, and implausible data points were excluded to ensure data integrity, yielding a standardized analytic cohort for the subsequent analyses. The analyses were performed using R version 4.3.0 (R Core Team, Vienna, Austria) and SAS version 8.3 (SAS Institute Inc., Cary, NC, USA). Statistical significance was set at a two-sided *P *value of <.05; for multiple comparisons, significance levels were adjusted using the Bonferroni correction.

## Results

###  Patient Characteristics

 Overall, 44 220 patients were enrolled. Table 1 outlines the patients’ baseline characteristics categorized by the three dental care settings. All groups contained more male than female patients. Overall, the average patient age was in the 70s, the income was highest in the fourthquartile, and the highest proportion of disabilities was “none.” Hospital-based dental clinics treated a higher proportion of patients with hypertension, diabetes, and dyslipidemia, and the lowest proportion of patients with a CCI score of 0 compared to private and group dental practices. Hospital-based dental clinics had the highest proportions of ex-smokers, smokers, and alcohol consumers compared to private and group dental practices. In all three groups, most patients did not exercise regularly during the week.

**Table 1:** Baseline Characteristics of the Study Population

**Variable**	**Private Dental Practices ** **(n = 40 502)**	**Group Dental Practices (n = 2629)**	**Hospital-Based Dental Clinics (n = 1089)**	***P*** ** Value**
Sex (%)				<.001
Male	22 100 (54.6)	1464 (55.7)	754 (69.2)	
Female	18 402 (45.4)	1165 (44.3)	335 (30.8)	
Age (y)	71.2 (4.9)	70.7 (4.8)	71.4 (4.7)	<.001
Income level (%)				<.001
1st Quartile	6401 (15.8)	378 (14.4)	145 (13.3)	
2nd Quartile	6826 (16.9)	403 (15.3)	176 (16.2)	
3rd Quartile	11 334 (28.0)	683 (26.0)	314 (28.8)	
4th Quartile	15 941 (39.4)	1165 (44.3)	454 (41.7)	
Disability (%)				<.001
No	35 400 (87.4)	2346 (89.2)	916 (84.1)	
Mild	796 (2.0)	50 (1.9)	41 (3.8)	
Severe	4306 (10.6)	233 (8.9)	132 (12.1)	
Residence (%)				.041
Rural	16 108 (39.8)	1049 (39.9)	392 (36.0)	
Urban	24 394 (60.2)	1580 (60.1)	697 (64.0)	
Hypertension (%)	28 143 (69.5)	1710 (65.0)	818 (75.1)	<.001
Diabetes (%)	10 492 (25.9)	615 (23.4)	331 (30.4)	<.001
Dyslipidemia (%)	23 375 (57.7)	1514 (57.6)	706 (64.8)	<.001
Osteoporosis (%)	5272 (13.0)	320 (12.2)	132 (12.1)	.327
CCI (%)				<.001
0	8967 (22.1)	618 (23.5)	176 (16.2)	
1	10 050 (24.8)	674 (25.6)	191 (17.5)	
2	8198 (20.2)	528 (20.1)	188 (17.3)	
≥3	13 287 (32.8)	809 (30.8)	534 (49.0)	
Body mass index (kg/m^2^)	24.4 (3.0)	24.2 (2.9)	24.6 (2.8)	<.001
Systolic blood pressure (mm Hg)	128.4 (14.4)	126.6 (14.0)	127.6 (14.6)	<.001
Diastolic blood pressure (mm Hg)	76.4 (9.4)	75.7 (9.1)	75.6 (9.4)	<.001
Fasting blood glucose (mg/dL)	105.6 (25.2)	104.8 (24.7)	107.4 (27.2)	.015
Hemoglobin (g/dL)	13.9 (1.4)	13.9 (1.4)	14.0 (1.5)	.008
Glomerular filtration rate (mL/min/1.73 m^2^)	79.9 (36.1)	79.6 (33.5)	79.6 (55.5)	.932
Smoking (%)				<.001
Non-smoker	27 431 (67.7)	1770 (67.3)	603 (55.4)	
Ex-smoker	10 009 (24.7)	674 (25.6)	408 (37.5)	
Smoker	3062 (7.6)	185 (7.0)	78 (7.2)	
Alcohol consumption (%)	13 340 (32.9)	850 (32.3)	399 (36.6)	.028
Regular exercise (%)				.041
No	23 740 (58.6)	1486 (56.5)	610 (56.0)	
1-2 times/week	5796 (14.3)	400 (15.2)	152 (14.0)	
3-4 times/week	5267 (13.0)	375 (14.3)	171 (15.7)	
5 times/week	5699 (14.1)	368 (14.0)	156 (14.3)	

Abbreviation: CCI, Charlson Comorbidity Index.

###  Association Between Dental Care Settings and the Risk of Dental Implant Failure 

Table 2 presents the associations between the risk of dental implant failure and the dental care setting. Dental implant failure occurred in 1042 of 40 502 of cases (14.22%) in private dental practices, 69 of 2629 cases (15.00%) in group dental practices, and 14 of 1089 cases (6.70%) in hospital-based dental clinics. In the Cox proportional hazards model, hospital-based dental clinics exhibited a substantially lower risk of dental implant failure than that in private and group dental practices.

**Table 2:** Association Between the Type of Dental Care Setting and Risk of Dental Implant Failure

**Group**	**Number**	**Events**	**Follow-up Duration (Person-Years)**	**Incidence Rate (Per 1000 Person-Years)**	**Crude HR** **(95% CIs,** ***P*** ** Value)**	**IPTW Adjusted HR** **(95% CIs,** ***P*** Value)*
Private dental practices (Reference)	40 502	1042	73 299.92	14.22	1 (Reference)	1 (Reference)
Group dental practices	2629	69	4601.03	15.00	1.05 (0.82-1.34, *P* = .707)	1.03 (0.91-1.15, *P* = .671)
Hospital-based dental clinics	1089	14	2089.02	6.70	0.48 (0.28-0.81, *P* = .006)	0.35 (0.30-0.41, *P* < .001)
Group dental practices (Reference)	2629	69	4601.03	15.00	1 (Reference)	1 (Reference)
Hospital-based dental clinics	1089	14	2089.02	6.70	0.46 (0.26-0.81, *P* = .007)	0.34 (0.20-0.58, *P* < .001)

Abbreviations: HR, hazard ratio; CIs, confidence intervals; IPTW, inverse probability of treatment weighting. * The model was adjusted for age, sex, income level, disability, residence, hypertension, diabetes, dyslipidemia, osteoporosis, CCI, smoking, alcohol consumption, regular exercise, body mass index, hemoglobin, and glomerular filtration rate.

 In the Cox proportional hazards model for private dental practices, the risk of dental implant failure was significantly lower in hospital-based dental clinics than that in private dental practices (crude HR: 0.48, 95% CI: 0.28–0.81). These results were reinforced by IPTW analysis (adjusted HR: 0.35, 95% CI: 0.30–0.41). The risk of dental implant failure did not differ significantly between private and group dental practices. However, considering the increasing emergence of group dental practices, we conducted an additional analysis to examine the potential differences between group dental practices and hospital-based dental clinics.

**Figure 2:**
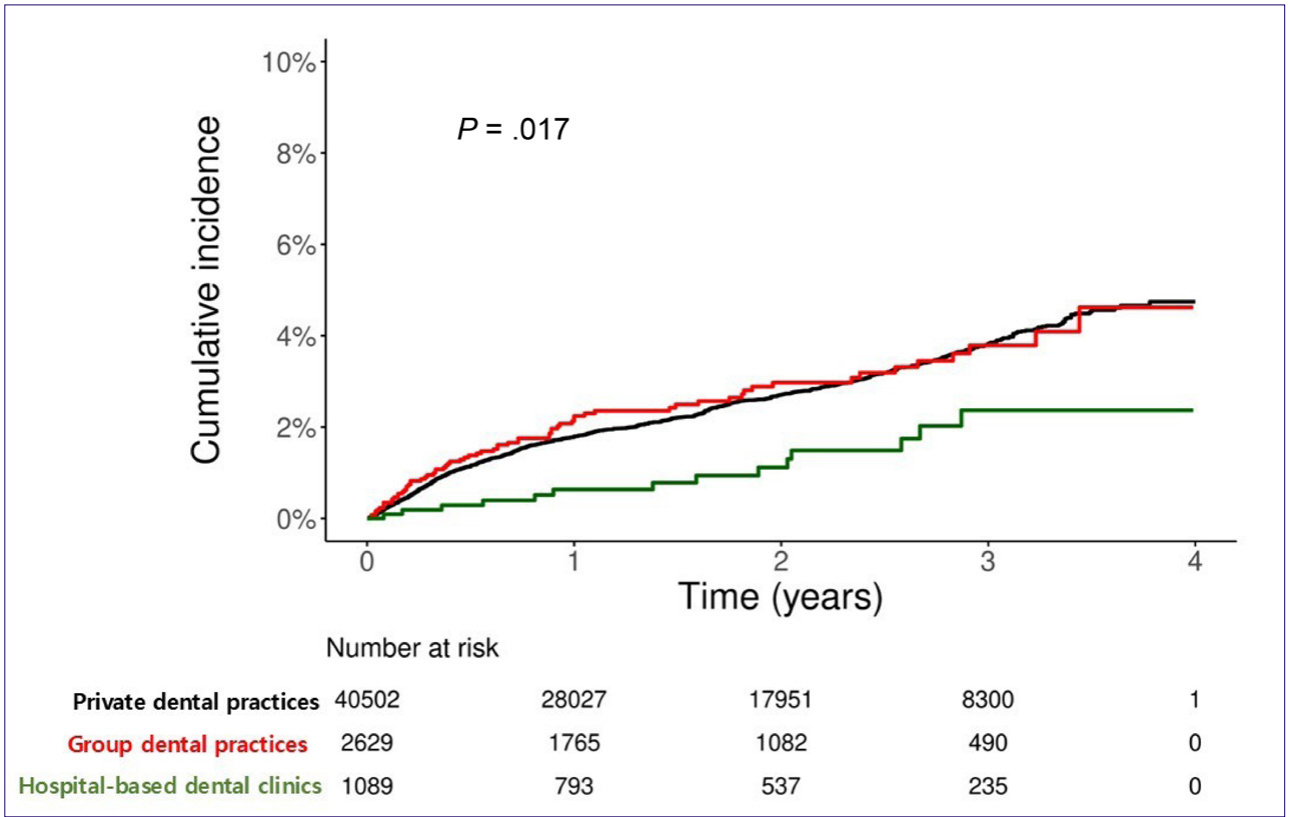
Kaplan–Meier Curve for the Association Between the Dental Care Setting and Dental Implant Failure.

 In the Cox proportional hazards model for group dental practices, the risk of dental implant failure was lower in hospital-based dental clinics than that in group dental practices (crude HR: 0.46, 95% CI: 0.26–0.81). These results were reinforced by IPTW analysis (adjusted HR: 0.34, 95% CI: 0.20–0.58). The Kaplan–Meier curve demonstrated a significant decrease in the probability of remaining disease-free in private dental practices and group dental practices compared with hospital-based dental clinics (log-rank test: *P* =.017; Figure 2).

###  Association Between Quartiles of Implant Procedural Counts in Private Dental Practices and the Risk of Dental Implant Failure 

**Figure 3:**
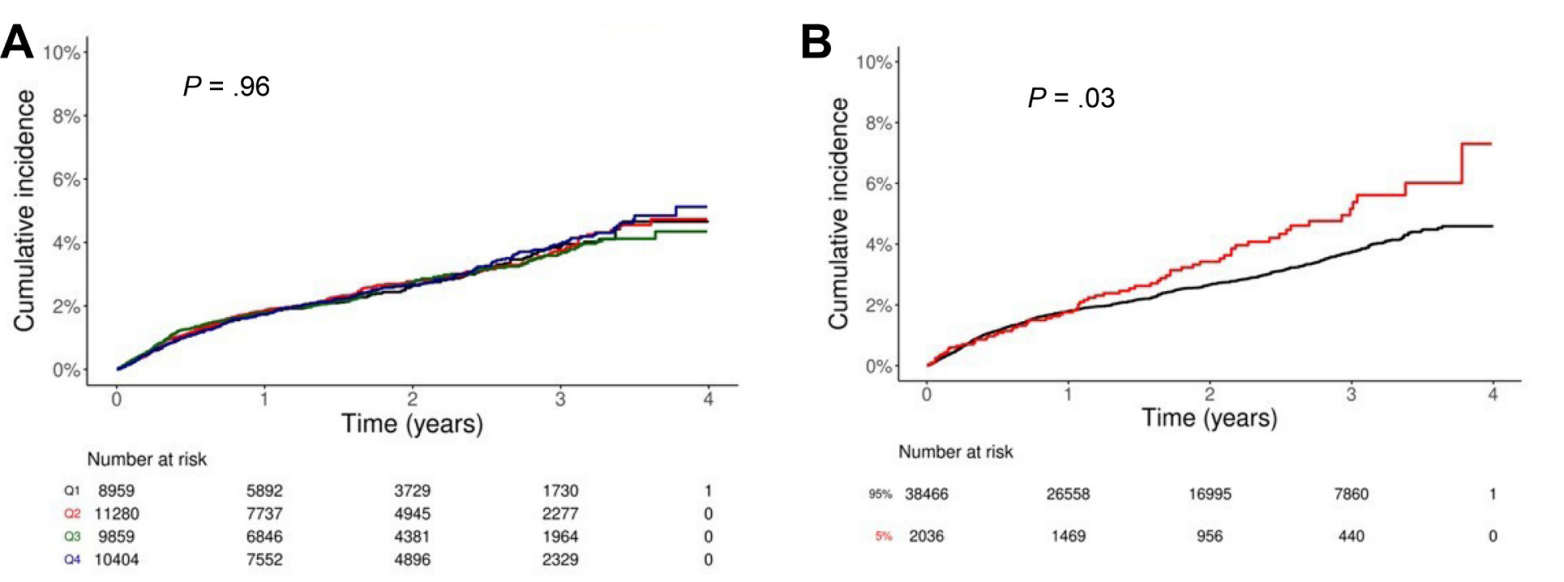
Kaplan–Meier Curves for the Association Between the Number of Implant Procedures Performed in Private Dental Practices (Quartiles and Top 5%) and Dental Implant Failure.

 Over 90% of all implant procedures were performed at private dental practices and were associated with a high risk of failure. To investigate whether the risk of failure varied with procedural volume, only private dental practices were categorized into four quartiles based on the number of procedures performed. Table S2 presents the confirmed sociodemographic characteristics, which were determined by dividing the private dental practices group into four quartiles based on the number of dental implant procedures performed in 4-year periods. An average of 2.1, 5.4, 10.4, and 33.6 dental implant procedures were performed in Q1, Q2, Q3, and Q4, respectively. The dental implant failure risk did not differ among the quartiles (Table 3), which was confirmed by Kaplan–Meier analysis with the log-rank test (*P* =.96; Figure 3A).

**Table 3:** Risk of Dental Implant Failure According to Quartiles of the Number of Dental Implant Procedures in Private Dental Practices

**Group**	**Number**	**Mean (SD) for Counts**	**Events**	**Follow-up Duration** **(Person-Years)**	**Incidence Rate** **(Per 1000 Person-Years)**	**Crude HR** **(95% CIs,** ***P*** ** Value)**	**IPTW Adjusted HR** **(95% CIs,** ***P *** Value)*
Quartile group							
Q1	8959	2.1 (0.8)	219	15 478.44	14.15	1 (Reference)	1 (Reference)
Q2	11 280	5.4 (1.1)	291	20 220.21	14.39	1.02 (0.86-1.22, *P* = .793)	1.04 (0.88-1.24, *P* = .632)
Q3	9859	10.4 (1.9)	248	17 814.90	13.92	0.99 (0.83-1.19, *P* = .942)	1.05 (0.88-1.26, *P* = .583)
Q4	10 404	33.6 (26.3)	284	19 786.37	14.35	1.03 (0.87-1.23, *P* = .714)	1.10 (0.92-1.31, *P* = .304)
Remaining 75%	30 098	6.1 (3.6)	758	53 513.55	14.16	1 (Reference)	1 (Reference)
Top 25%	10 404	33.6 (26.3)	284	19 786.37	14.35	1.03 (0.90-1.18, *P* = .707)	1.09 (0.96-1.22, *P* = .177)
Remaining 90%	36 322	8.4 (6.4)	912	65 148.96	14.00	1 (Reference)	1 (Reference)
Top 10%	4180	53.8 (31.9)	130	8150.96	15.95	1.16 (0.96-1.39, *P* = .121)	1.23 (1.09-1.38, *P* < .001)
Remaining 95%	38 466	9.9 (8.6)	972	69 438.32	14.00	1 (Reference)	1 (Reference)
Top 5%	2036	74.8 (34.8)	70	3861.60	18.13	1.31 (1.03-1.67, *P* = .030)	1.38 (1.23-1.54, *P* < .001)

Abbreviations: SD, standard deviation; HR, hazard ratio; CIs, confidence intervals; IPTW, inverse probability of treatment weighting. * The model was adjusted for age, sex, income level, disability, residence, hypertension, diabetes, dyslipidemia, osteoporosis, CCI, smoking, alcohol consumption, regular exercise, body mass index, hemoglobin, and glomerular filtration rate.

 Our subsequent analysis only included the top 25% of dental implant procedures performed at private dental practices. The top 25%, 10%, and 5% of private dental practices performed an average of 33.6, 53.8, and 74.8 procedures, with attendant dental implant failure rates of 14.35%, 15.95%, and 18.13%, respectively. IPTW analysis revealed that the top 10% private dental practices had a 1.23 (95% CI: 1.09–1.38) higher risk of dental implant failure compared with the remaining 90%. Additionally, the top 5% had a 1.31 (95% CI: 1.03–1.67) higher risk of dental implant failure per the Cox analysis, and a 1.38 (95% CI: 1.23–1.54) higher risk per IPTW analysis compared with the remaining 95%. Kaplan–Meier curves showed a significantly higher probability of dental implant failure in the top 5% compared with the remaining 95% (log-rank test: *P* =.03; Figure 3B).

## Discussion

 This study examined the association between the dental care setting and the risk of dental implant failure using the NHIS-HEALS database, revealing that the setting was significantly associated with the rate of implant failure. To our knowledge, this is the first study to analyze dental implant failure with respect to the dental care setting. Although previous studies have explored implant complications and causes of failure, they were largely multicenter retrospective analyses or short-term follow-up studies conducted in a limited number of large institutions. Research assessing implant failure across various institutions, ranging from private dental practices to hospital-based dental clinics, is scarce.

 A key finding of this study was the negative correlation between the dental care setting and the dental implant failure risk. Specifically, private and group dental practices exhibited significantly higher failure rates than hospital-based dental clinics. These findings align with those of Yoon et al.^20^ Our study expands on prior research by incorporating re-implantation and dental implant removal, offering a more comprehensive assessment of implant failure. The robustness of our findings was further enhanced through IPTW analysis, which was conducted to balance the confounding variables across dental care settings.

 We found that 91.59% of the reimbursed dental implant procedures were performed in private dental practices. Although most implants were placed in private dental practices, the failure rates were similar between private and group dental practices, suggesting that a high procedural volume alone does not account for implant failure, and that other factors must be considered. The heightened failure rates in private and group dental practices are likely influenced by intense market competition. Unlike hospital-based dental clinics, where implant pricing is stable, private and group dental practices often use aggressive pricing strategies for reimbursed and non-reimbursed implants. This trend has raised patient safety concerns, prompting public awareness campaigns on low-cost and high-volume dental practices. For example, according to 2023 data from the Health Insurance Review and Assessment Service, 75.5% of private and group dental practices opened and subsequently closed, leading to numerous abandoned implant treatments and greater patient harm.^23,24^

 The success of dental implants is largely contingent on the oral surgeon’s expertise,^25^ and the surgical team’s proficiency and the institution’s available medical resources significantly affect outcomes. Although differences in implant failure rates according to the dental care setting cannot be explained solely by expertise, it is necessary to consider why failures are particularly high in private and group dental practices. In private dental practice, a single dentist is typically responsible for all procedures, which limits the time available for each patient. Corporate dental practices in Korea—often organized as group dental practices—prioritize high-volume procedures, potentially compromising personalized treatment planning and patient outcomes. The optimal implantation timing must be adjusted according to the patient’s alveolar bone condition; however, patient-driven pressure to accelerate the second- and third-stage procedures or the pursuit of experimental techniques can increase the risk of failure. Moreover, the growing trend of corporate dental practices offering extended operating hours and year-round services may further degrade treatment quality owing to clinician fatigue.^26^ Nevertheless, the utilization rate of private and group dental practices is bound to be higher because they are more accessible than hospital-based dental clinics, and the additional rate for each type of dental care setting is low; therefore, the patient’s out-of-pocket expenses are relatively low. Patients should comprehensively consider the dentist’s experience and the dental care setting’s capabilities, multidisciplinary approach, and response to emergencies when selecting a dental implant procedure.

 Alternatively, studies on the volume–outcome relationship of hospitals have shown that hospitals that treat patients with specific diseases or perform specific surgeries or procedures in large volumes have lower mortality rates.^27,28^ Similarly, dental implant surgery, which requires significant technical expertise, is influenced by procedural volume. Yoon et al^20^ analyzed implant failure rates by implant procedure frequency quartile and reported that the higher the procedure frequency, the better the treatment outcome. Another study reported that clinicians who placed at least 50 implants had significantly lower failure rates.^29^ However, these studies did not distinguish outcomes by dental care setting. Our study examined volume–outcome relationships, specifically in private dental practices, where most implants are placed. We found that the failure of dental implants was higher in the top 5% than in the top 10%. These results differ from the existing volume outcomes, and as explained previously, it can be assumed that there is a high possibility of failure due to excessive competition in private dental practices. Intense price competition can drive patients toward low-cost providers where per-patient procedural investment is minimal, potentially contributing to increased implant failures. This cost-cutting cycle ultimately shifts the risks and burdens to the patients. Consequently, when selecting a dental provider, patients should consider the procedural volume, case complexity, clinician experience, and institutional resources. In addition, if dentists set their own limits for the number of procedures performed per day in elderly patients, patients with complications, or cases with high surgical difficulty, detailed treatment plans and more meticulous procedures will be possible through sufficient case analysis.

 Patient characteristics varied by the dental care setting. Compared with private and group dental practices, hospital-based dental clinics treated more patients with severe disabilities, multiple comorbidities (excluding osteoporosis), and a CCI score ≥3. Additionally, a greater percentage of former and current smokers, as well as alcohol consumers, sought treatment in hospital-based dental clinics. Although systemic diseases,^30^ smoking,^31^ and alcohol consumption^32^ are recognized as risk factors for implant failure, the lower failure rates observed in hospital-based dental clinics warrant careful interpretation. Hospital-based dental clinics typically have specialized dental departments that provide a structured and multidisciplinary approach to implant procedures. Moreover, cases with high surgical complexity may benefit from enhanced procedural planning and emergency response capabilities. However, concluding that hospital-based dental clinics always yield superior outcomes would be an oversimplification because many interrelated factors influence treatment success. Therefore, a careful assessment of failure rates is necessary when considering dental implant procedures in dental care settings.

 This study had several limitations. First, the NHIS-HEALS database included only reimbursed dental implants and excluded non-reimbursed cases. Consequently, the recorded failure rates, including re-implantation (reimbursed implants) and removal (reimbursed and non-reimbursed implants), may have been underestimated. Second, key clinical variables affecting implant failure, such as alveolar bone height and quality, and genetic factors, were not available in the claims data, limiting our ability to assess surgical difficulty. Additionally, implant-specific factors (eg, fixture length and diameter, implant system) that influence primary stability could not be analyzed. Information regarding the clinician’s technique or surgical protocol was also not captured. Third, the identification of the outcome events may not have been precise. Non-reimbursed implant failures may be underreported in certain private and group dental practices due to billing concerns, and patients in some institutions may be less likely to return for follow-up treatment, potentially leading to underestimation of failure rates. Finally, because reimbursement for dental implants has only recently been introduced in Korea, our study included only short-term follow-up data. Long-term studies are needed to elucidate the determinants of implant failure. Collectively, these limitations underscore the need for future studies that include both reimbursed and non-reimbursed cases and incorporate detailed clinical variables with long-term follow-up. Well-designed prospective studies are essential to clarify the determinants of implant failure and inform clinical and policy decisions.

## Conclusions

 This study identified a significant association between the dental care setting and the risk of dental implant failure, finding that failure rates are lower in hospital-based dental clinics than those in private and group dental practices. These results highlight the need for more systematic surgical protocols and comprehensive follow-up care in private and group dental practices to enhance implant stability and patient safety.

## Disclosure of artificial intelligence (AI) use

 Not applicable.

## Ethical issues

 This study was approved by the Institutional Review Board of Youngsan University (IRB No. YSUIRB-202412-HR-167-02) conducted in accordance with the Code of Ethics of the World Medical Association (Declaration of Helsinki) and waived the need for the informed consent.

## Conflicts of interest

 Authors declare that they have no conflicts of interest.

## Supplementary files


Supplementary file 1 contains Figure S1 and Tables S1-S2.
