# Supplementary material for: Association Between the Type of Dental Care Setting and the Risk of Dental Implant Failure in Korea: A Retrospective Nationwide Population-Based Cohort Study
Source: Int J Health Policy Manag. 2025 Dec 28;14:9238. doi: 10.34172/ijhpm.9238 (PMC12958168; doi:10.34172/ijhpm.9238)
Supplement: Supplementary file 1 — contains Figure S1 and Tables S1-S2. [file ijhpm-14-9238-s001.pdf]

Article title: Association Between the Type of Dental Care Setting and the Risk of Dental Implant Failure in Korea: A Retrospective Nationwide Population-Based Cohort Study

Journal name: International Journal of Health Policy and Management (IJHPM)

Authors' information: Yu-Rin Kim[1]¶, Seon-Rye Kim[2]¶, Minkook Son[3,4]*

[1]Department of Dental Hygiene, Silla University, Busan, Republic of Korea.

[2]Institute of Health Medical Education Convergence Research, Kangwon National University, Gangwon-do, Republic of Korea.

[3]Department of Physiology, Dong-A University College of Medicine, Busan, Republic of Korea.
[4]Department of Data Sciences Convergence, Dong-A University Interdisciplinary Program, Busan, Republic of Korea.

***Correspondence to:** Minkook Son; Email: physionet@dau.ac.kr

¶ Both authors contributed equally to this paper.

Citation: Kim YR, Kim SR, Son M. Association between the type of dental care setting and the risk of dental implant failure in Korea: A retrospective nationwide population-based cohort study. Int J Health Policy Manag.2025;14:9238. doi:10.34172/ijhpm.9238

Supplementary file 1

Table S1: Dental implant (per tooth) procedure code

Table S2: Baseline characteristics by quartile group for dental implant procedures in private dental practices

Figure S1: Distributions of propensity scores before and after IPTW across groups

Table S1. Dental implant (per tooth) procedure code

	ICD-10	Procedure content	Dental care settings	Procedure codes
**Dental implant users** (Stage 2 Hospital-based dental clinics UB115, UB117 completed case)	K081	Stage 1<br>Diagnosis and treatment plan	Private dental practices<br>Group dental practices	UB111<br>UB112, UB113, UB116, UB118
		**Stage 2**<br>**Dental implant fixture (body) placement surgery**	**Private dental practices**	**UB121**
			**Group dental practices**	**UB122, UB123, UB126, UB128**
			**Hospital-based dental clinics**	**UB125, UB127**
		Stage 3	Private dental practices	UB131

	Dental implant prosthesis restoration	Group dental practices	UB132, UB133, UB136, UB138
		Hospital-based dental clinics	UB135, UB137
**Dental implant failure**	Definition 1<br>Dental Implant reimplantation (only reimbursable dental implants)	Private dental practices	UB121002
		Group dental practices	UB122002<br>UB123002,<br>UB126002,<br>UB128002
		Hospital-based dental clinics	UB125002,<br>UB127002
	Definition 2<br>Dental implant removal surgery (includes both reimbursable and non-reimbursable dental implants)	Simple (when the fixture was unstable due to osseointegration failure)	U4981
		Complex (when there was no mobility in the dental implant fixture, but the implant fixture was removed using a trephine burr or a separate dedicated removal kit due to periimplantitis, fracture, or nerve damage)	U4982

Table S2. Baseline characteristics by quartile group for dental implant procedures in private dental practices

**Variable**		**1st quartile (n = 8959)**	**2nd quartile (n = 11280)**	**3rd quartile (n = 9859)**	**4th quartile (n = 10404)**	**P-value**
**Sex (%)**	Male	5082 (56.7)	6205 (55.0)	5293 (53.7)	5520 (53.1)	<0.001
	Female	3877 (43.3)	5075 (45.0)	4566 (46.3)	4884 (46.9)	
**Age (years)**		70.8 (4.8)	71.0 (4.9)	71.4 (5.0)	71.5 (5.0)	<0.001
**Income level (%)**	1st quartile	1544 (17.2)	1866 (16.5)	1470 (14.9)	1521 (14.6)	<0.001
	2nd quartile	1533 (17.1)	1965 (17.4)	1609 (16.3)	1719 (16.5)	
	3rd quartile	2409 (26.9)	3101 (27.5)	2840 (28.8)	2984 (28.7)	
	4th quartile	3473 (38.8)	4348 (38.5)	3940 (40.0)	4180 (40.2)	
**Disability (%)**	No	7936 (88.6)	9962 (88.3)	8545 (86.7)	8957 (86.1)	<0.001
	Mild	149 (1.7)	202 (1.8)	218 (2.2)	227 (2.2)	
	Severe	874 (9.8)	1116 (9.9)	1096 (11.1)	1220 (11.7)	
**Residence (%)**	Rural	2346 (26.2)	3561 (31.6)	4216 (42.8)	5985 (57.5)	0.001
	Urban	6613 (73.8)	7719 (68.4)	5643 (57.2)	4419 (42.5)	
**Hypertension (%)**		6177 (68.9)	7815 (69.3)	6851 (69.5)	7300 (70.2)	<0.001
**Diabetes (%)**		2264 (25.3)	2927 (25.9)	2517 (25.5)	2784 (26.8)	<0.001
**Dyslipidemia (%)**		5200 (58.0)	6551 (58.1)	5621 (57.0)	6003 (57.7)	<0.001

**Osteoporosis (%)**		1044 (11.7)	1399 (12.4)	1361 (13.8)	1468 (14.1)	0.187
**Charlson comorbidity index (%)**	0	2237 (25.0)	2533 (22.5)	2124 (21.5)	2073 (19.9)	<0.001
	1	2259 (25.2)	2888 (25.6)	2441 (24.8)	2462 (23.7)	
	2	1736 (19.4)	2317 (20.5)	2066 (21.0)	2079 (20.0)	
	≥ 3	2727 (30.4)	3542 (31.4)	3228 (32.7)	3790 (36.4)	
**Body mass index (kg/m2)**		24.5 (3.0)	24.4 (3.0)	24.4 (2.9)	24.4 (3.0)	0.150
**Systolic blood pressure (mmHg)**		128.3 (14.3)	128.2 (14.5)	128.4 (14.4)	128.6 (14.6)	0.351
**Diastolic blood pressure (mmHg)**		76.4 (9.3)	76.2 (9.4)	76.5 (9.4)	76.6 (9.3)	0.027
**Fasting blood glucose (mg/dL)**		105.1 (24.5)	105.7 (24.4)	105.5 (26.0)	106.1 (25.7)	0.033
**Hemoglobin (g/dL)**		14.0 (1.4)	13.9 (1.4)	13.8 (1.5)	13.8 (1.5)	<0.001
**Glomerular filtration rate (mL/min/1.73 m^2)**		80.3 (39.7)	80.0 (36.4)	79.8 (35.2)	79.3 (33.1)	0.265
**Smoking (%)**	Nonsmoker	5809 (64.8)	7530 (66.8)	6741 (68.4)	7351 (70.7)	<0.001
	Ex-smoker	2436 (27.2)	2856 (25.3)	2401 (24.4)	2316 (22.3)	
	Smoker	714 (8.0)	894 (7.9)	717 (7.3)	737 (7.1)	
**Alcohol consumption (%)**		3231 (36.1)	3818 (33.8)	3157 (32.0)	3134 (30.1)	<0.001
**Regular exercise (%)**	No	5003 (55.8)	6407 (56.8)	5934 (60.2)	6396 (61.5)	<0.001
	1-2 times/week	1335 (14.9)	1689 (15.0)	1352 (13.7)	1420 (13.6)	

Variable		1st quartile (n = 8959)	2nd quartile (n = 11280)	3rd quartile (n = 9859)	4th quartile (n = 10404)	P-value
	3-4 times/week	1302 (14.5)	1552 (13.8)	1244 (12.6)	1169 (11.2)	
	5 times/week	1319 (14.7)	1632 (14.5)	1329 (13.5)	1419 (13.6)	
**Dental procedural counts**		2.1 (0.8)	5.4 (1.1)	10.4 (1.9)	33.6 (26.3)	<0.001

Figure S1. Distributions of propensity scores before and after IPTW across groups

1) Private dental practices vs Group dental practices

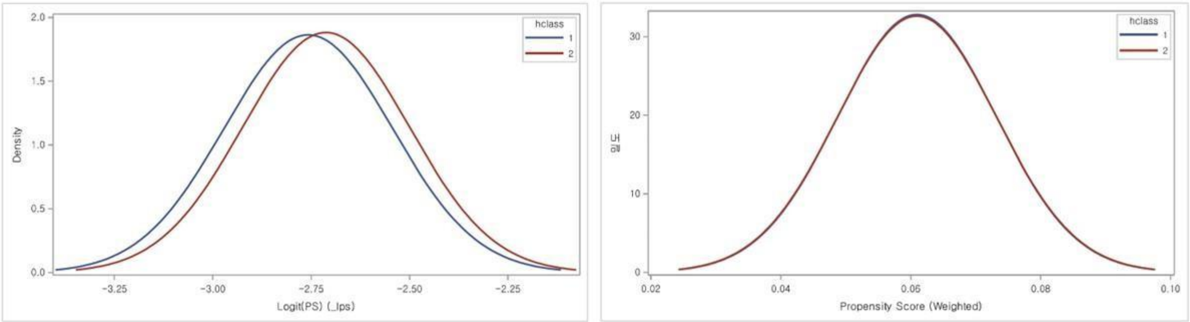

Before IPTW After IPTW

2) Private dental practices vs Hospital-based dental clinics

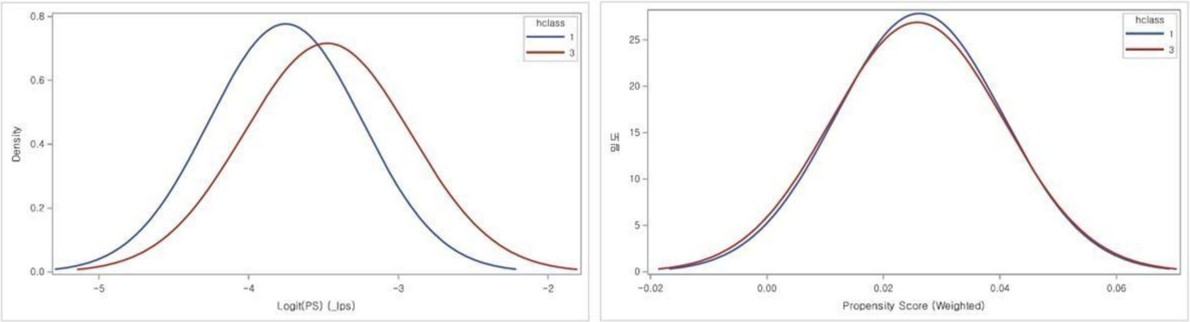

Before IPTW After IPTW

3) Group dental practices vs Hospital-based dental clinics

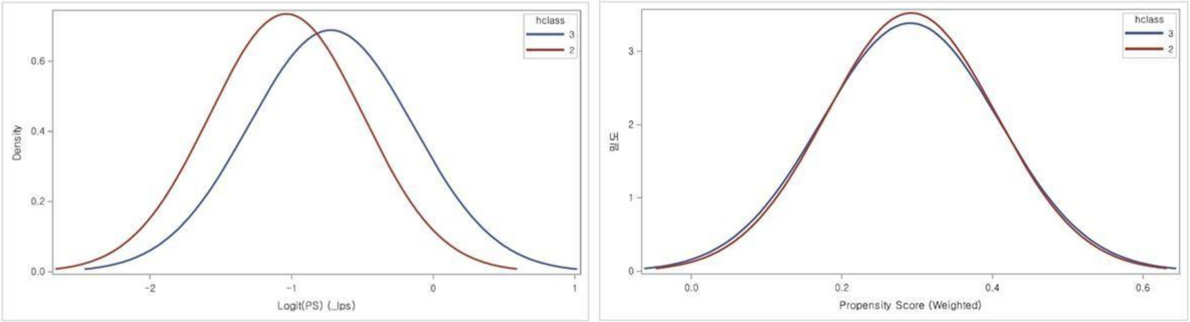

Before IPTW After IPTW
